# Immune in myocardial ischemia/reperfusion injury: potential mechanisms and therapeutic strategies

**DOI:** 10.3389/fimmu.2025.1558484

**Published:** 2025-05-08

**Authors:** Xiaoyu Xuan, Jilin Fan, Jingyi Zhang, Ming Ren, Limin Feng

**Affiliations:** ^1^ College of Traditional Chinese Medicine, Tianjin University of Traditional Chinese Medicine, Tianjin, China; ^2^ Department of Rehabilitation, The Affiliated Hospital of Binzhou Medical University, Binzhou, Shandong, China; ^3^ Department of Traditional Chinese Medicine, The Second Affiliated Hospital of Shandong First Medical University, Shandong, Taian, China; ^4^ Baokang Hospital Affiliated to Tianjin University of Traditional Chinese Medicine, Tianjin, China; ^5^ The Second Affiliated Hospital of Tianjin University of Traditional Chinese Medicine, Tianjin, China; ^6^ Tianjin Binhai New Area Traditional Chinese Medicine Hospital, Tianjin, China

**Keywords:** myocardial ischemia/reperfusion injury, myocardial infarction, immune cell, cell death, immunotherapy

## Abstract

Myocardial infarction (MI), which is characterized by high morbidity and mortality, is a serious threat to human life and health, and timely reperfusion therapy to save ischemic myocardium is currently the most effective intervention. Although reperfusion therapy effectively restores coronary blood flow and maximally limits the infarct size, it triggers additional cell death and tissue damage, which is known as myocardial ischemia/reperfusion injury (MIRI). Multiple immune cells are present in the reperfusion area, executing specific functions and engaging in crosstalk during diverse stages, constituting a complex immune microenvironment involved in tissue repair and regeneration after MIRI. Immunotherapy brings new hope for treating ischemic heart disease by modulating the immune microenvironment. In this paper, we explore the regulatory roles of various immune cells during MIRI and the close relationship between different cell deaths and the immune microenvironment. In addition, we present the current status of research on targeting the immune system to intervene in MIRI, with the expectation of providing a basis for achieving clinical translation.

## 1 Introduction

Disability and death caused by acute myocardial infarction (AMI) seriously affect the quality of life and health of human beings, and early reperfusion therapy to restore ischemic myocardial blood supply is currently the main therapeutic method (1). Percutaneous coronary intervention (PCI), as the preferred reperfusion therapy strategy, has greatly reduced the mortality rate of patients and has brought about remarkable improvements in the treatment and management of AMI (2). However, despite the reliable efficacy of reperfusion therapy in saving ischemic myocardium and restoring cardiac function, the process of reestablishing myocardial blood flow in the diseased myocardium can lead to myocardial structural and functional dysfunctions, aggravate the degree of MI, and even lead to a serious complication, namely myocardial ischemia/reperfusion injury (MIRI) (3). Clinical manifestations include myocardial stunning, no-reflow phenomenon, arrhythmia, and fatal reperfusion injury, the latter two of which are irreversible and can worsen the patient’s condition or even lead to death (4). The pathological mechanism of MIRI is complex. Research evidence suggests that it involves oxidative stress, intracellular calcium overload, mitochondrial dysfunction, energy metabolism disruption, inflammatory response, ferroptosis, autophagy, pyroptosis, and many other biological processes (5).

The immune system plays an important role in the repair and regeneration of cardiac tissue. Immune cells that reside in and infiltrate cardiac tissue are involved in the maintenance of cardiac homeostasis and repair function. During the pathological process of MIRI, diverse subpopulations of immune cells are present in the reperfusion area, which undergo dynamic changes at different time points and exhibit function heterogeneously at various stages of the disease (6). The main immune cell types in the heart are macrophages, neutrophils, dendritic cells, T-cells, B-cells, innate lymphocytes, and mast cells, and different subpopulations of resident and recruited immune cells have specific functions (7). Among them, macrophages are the most abundant immune cells in both the homeostatic and damaged states of the heart, and play an important role in cardiac development, maintaining homeostasis, and promoting repair after injury (8). However, the first immune cells to massively infiltrate the damaged area after the onset of MIRI are not macrophages but neutrophils. The function of neutrophils remains unclear. They can initiate and amplify the acute inflammatory response and also play a beneficial role in the process of inflammation abatement and cardiac healing (9). Both T cells and B cells among adaptive immune cells are involved in regulating wound healing and tissue remodeling after MIRI. For example, regulatory T cells (Tregs), which accumulate abundantly in the damaged myocardium, increase collagen content and promote scar maturation by expressing Sparc (a matricellular protein), which in turn prevents cardiac rupture and exerts a cardioprotective effect (10). Various immune cells during MIRI can interact and crosstalk with each other, constituting an intricate immune microenvironment. In addition, cardiomyocyte death is a key pathological aspect of MIRI, and multiple forms of cell death such as ferroptosis, apoptosis, and pyroptosis are closely related to the progression of MIRI (11). It has been reported that immune cells can interact with ferroptosis (12). It is evident that the immune response is highly involved in the pathological process of MIRI, and immunotherapy may be a promising treatment for MIRI.

In this paper, we reviewed the initiation of immune response during MIRI and the important role of different immune cells in cardiac homeostasis and injury repair. Furthermore, we explored the close relationship between multiple forms of cell death during MIRI and the immune system. Additionally, we summarized the current research progress in targeting the immune response for the treatment of MIRI. In recent years, researchers have designed a large number of novel biomaterials to improve drug delivery efficiency and therapeutic efficacy, yet there are almost no relevant reviews; therefore, in this paper, we describe the latest evidence of biomaterials for the treatment of MIRI through modulation of immunity, to provide a reference for clinical treatment. In this study, articles in PubMed and Web of Science were searched independently or in combination using the following keywords: immunity, myocardial ischemia/reperfusion injury, macrophage, neutrophil, dendritic cell, innate lymphocyte, T cell, B cell, ferroptosis, pyroptosis, immunotherapy.

## 2 Initiation of immune response in MIRI

**Figure 1:**
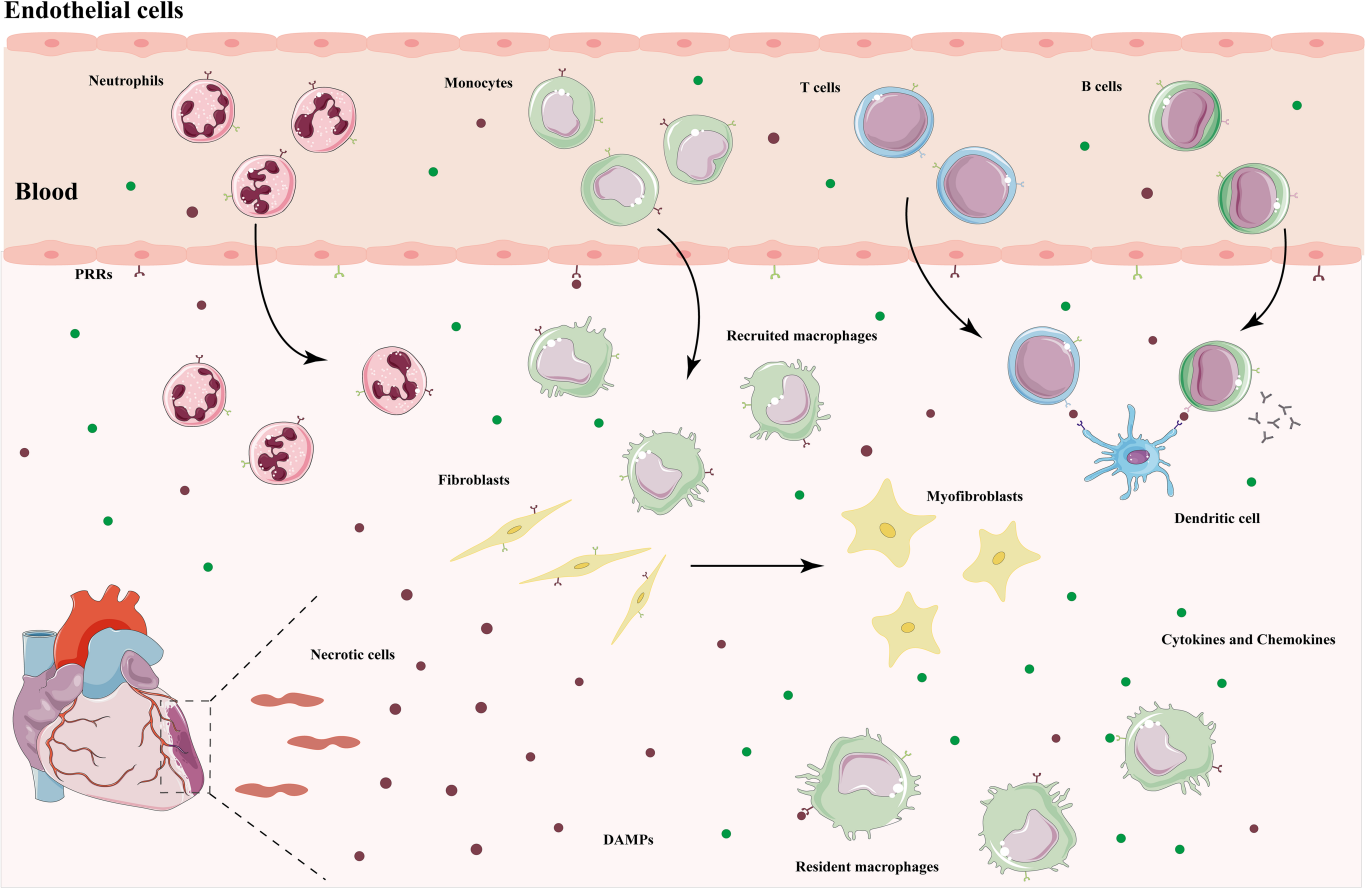
Immune response after myocardial ischemic injury. DAMPs released by necrotic cardiomyocytes bind to PRRs to initiate an immune response. Resident macrophages produce pro-inflammatory cytokines and chemokines, which recruit monocytes and neutrophils to the injured area to remove cellular debris and remodel the extracellular matrix. Dendritic cells present antigens to lymphocytes to activate an adaptive immune response. After entering the repair phase, immune cells transform into a repair phenotype and secrete anti-inflammatory cytokines to mediate the resolution of inflammation. Fibroblasts activate into myofibroblasts, leading to scar formation. DAMPs, damage-associated molecular patterns; PRRs, pattern recognition receptors.

The immune response plays an important role in the pathogenesis of MIRI, characterized by the recruitment and activation of innate and adaptive immune cells, and sterile inflammation occurs in the ischemic myocardium and surrounding tissues (**Figure 1**). The injury and death of cardiomyocytes resulting from ischemia and reperfusion lead to the release of damage-associated molecular patterns (DAMPs), which trigger a complex signaling cascade in the absence of pathogen invasion, initiating a strong inflammatory response and exacerbating cardiac injury (13). After reperfusion therapy, endogenous molecules such as high mobility group box 1 (HMGB-1), heat shock proteins (HSPs), hyaluronic acid, mitochondrial DNA (mtDNA), circulating extracellular RNA (exRNA) are released into the outside of the cell, and these intracellular components are known as DAMPs (14). Pattern recognition receptors (PRRs) are risk sensors for innate immune responses and mainly consist of Toll-like receptors (TLRs), NOD-like receptors (NLRs), C-type lectin receptors (CLRs), and RIG-I-like receptors (RLRs), which respond to danger signals and initiate immune responses by binding to DAMPs (15).

TLRs are the first family of PRRs to be recognized in humans and the most intensively studied. They activate the innate immune system by triggering downstream signaling through the recognition of DAMPs and are also an important bridge between innate and adaptive immunity (16). TLRs are not only present in a variety of immune cells, but also expressed in cardiovascular cells, such as cardiomyocytes, endothelial cells, and smooth muscle cells. Chronic low-grade inflammation resulting from sustained activation of TLRs accelerates cardiomyocyte death and adverse cardiac remodeling (17). Among them, TLR2 and TLR4 are highly expressed in cardiac tissues, and their binding to ligands prompt the translocation of nuclear factor-kappa B (NF-κB) and interferon regulatory factor (IRF) to the nucleus to initiate an inflammatory response, release inflammatory cytokines, and mediate cardiac injury (18). NLRP3 inflammasomes are major members of the NLR family, and the pathogenesis of MIRI is closely related to the initiation and activation of NLRP3 inflammasomes. During reperfusion, endogenous cytokine release, massive reactive oxygen species (ROS) production, calcium overload, and endothelial dysfunction all contribute to the formation and activation of NLRP3 inflammasomes, followed by IL-1β and IL-18 production as well as pyroptosis involved in the progression of MIRI (19). Pro-inflammatory cytokines and chemokines expressed by the cardiovascular system cells and cardiac resident immune cells recruit neutrophils, monocytes, and macrophages to the injury site, further activating the immune system and amplifying the inflammatory response (20). In addition, endothelial dysfunction is manifested by decreased NO production and increased expression of adhesion factors, which contribute to the adhesion and infiltration of neutrophils and monocytes (3). Thus, in the early stages of MIRI, DAMPs bind to PRRs to activate downstream signal transduction, upregulate inflammatory cytokines and chemokines, and in turn recruit a variety of innate immune cells to reach the injured area and initiate an immune response.

## 3 MIRI and immune microenvironment

**Figure 2:**
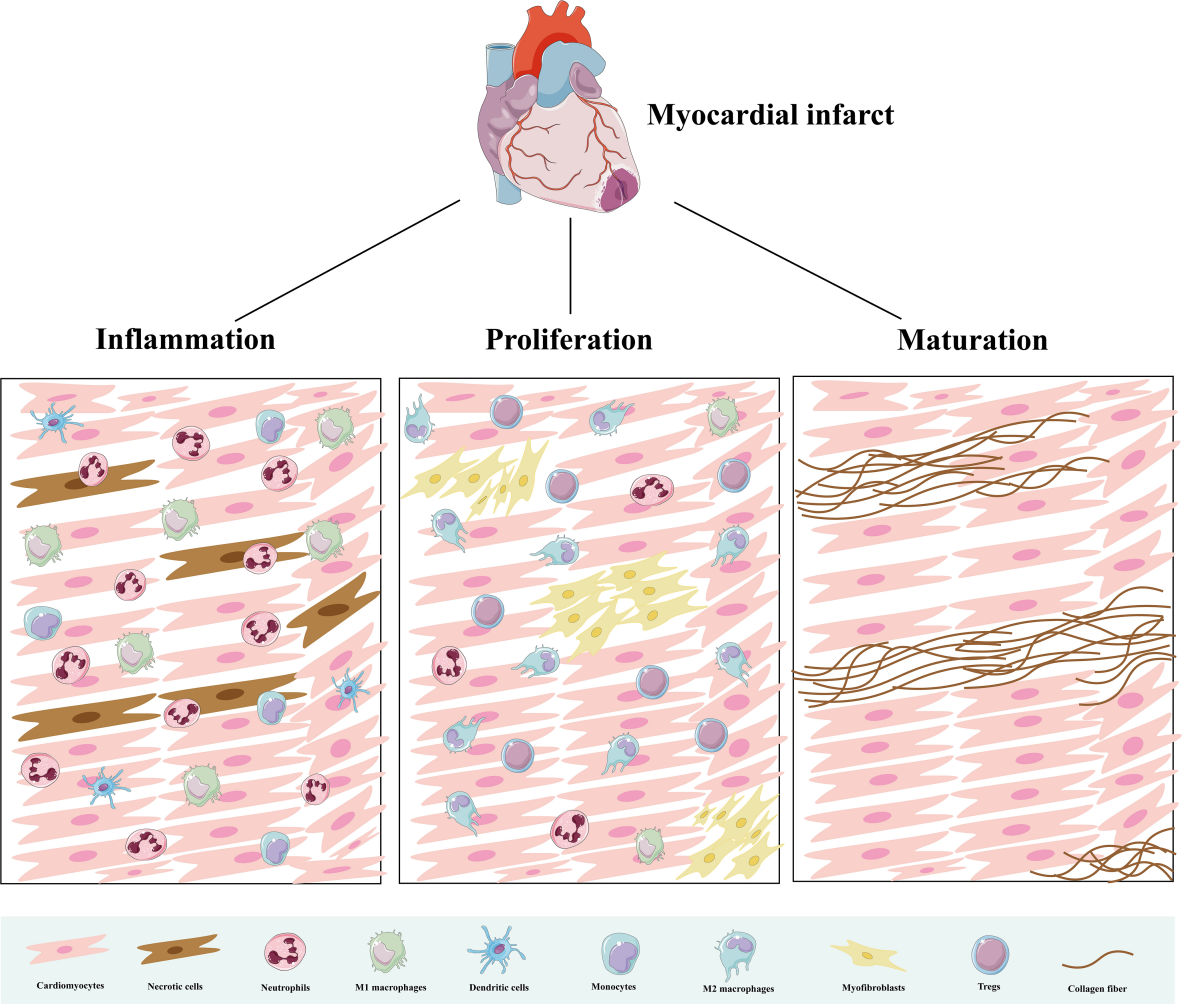
Immune cells in three pathological stages after AMI. During the inflammatory phase, immune cells clear dead cells and initiate an inflammatory response. During the proliferative phase, they promote angiogenesis and fiber repair. During the mature phase, immune cells decrease and collagen deposition forms mature scars.

**Figure 3:**
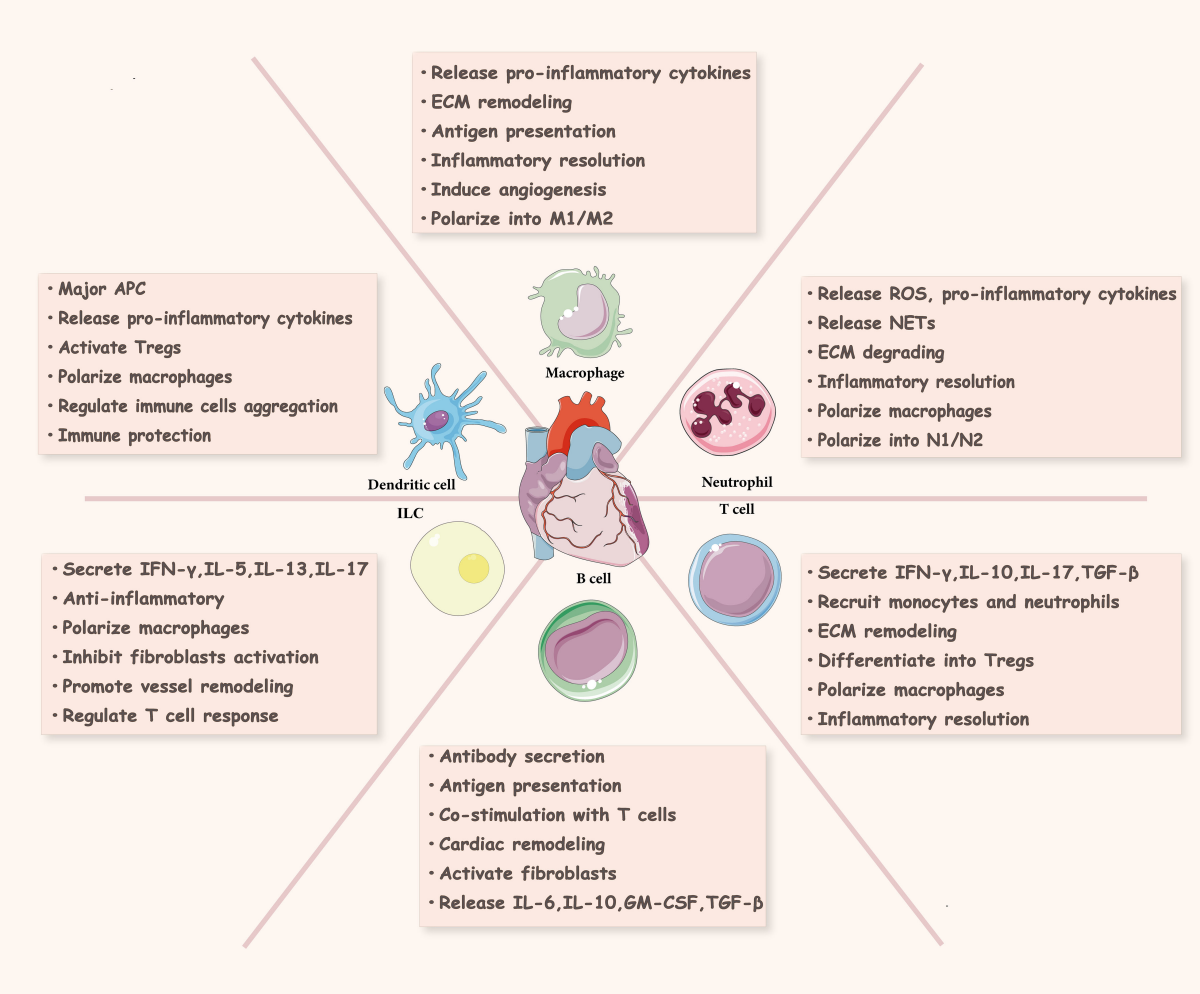
Immune cells in cardiac injury and repair. Innate immune cells (neutrophils, macrophages, dendritic cells, innate lymphocytes) and adaptive immune cells (T cells, B cells) in the ischemic heart are involved in cardiac tissue repair and regeneration. ROS, reactive oxygen species; NETs, neutrophil extracellular traps; ECM, extracellular matrix; APC, antigen presenting cell; ILC, innate lymphoid cell; INF-γ, interferon γ; GM-CSF, granulocyte-macrophage colony stimulating factor; TGF-β, transforming growth factor-β; Tregs, regulatory T cells.

Immune cells are the main component supporting the functioning of the immune system, and their repair process of MIRI undergoes three stages: inflammation, proliferation, and maturation (21) (**Figure 2**). There are various innate and adaptive immune cells in the heart, including immune cell populations that reside permanently in the heart and peripheral immune cells and their precursors recruited to the heart (7). Various immune cells perform specific functions and interact with each other at different stages to produce pro- or anti-inflammatory cytokines, modulate inflammation, cardiomyocyte proliferation, fibrosis, as well as extracellular matrix formation, and influence wound healing and scar formation to maintain cardiac structure and function (**Figure 3**).

### 3.1 Innate immunity

#### 3.1.1 Neutrophils

After the occurrence of AMI, neutrophils will rapidly accumulate in large numbers on the damaged myocardial tissue, the infiltration of neutrophils reaches the peak on days 1–3 and rapidly returns to a lower level on days 5-7 (9). Neutrophils are mainly involved in the early pathological process of reperfusion injury. In the injured area, neutrophils release large amounts of ROS, pro-inflammatory cytokines, chemokines, proteases, and so on, which promote the recruitment of more immune cells and exacerbate the inflammatory response as well as tissue damage. Evidence suggests that the alarm proteins S100A8 and S100A9 released by neutrophils can bind to TLR4, trigger the assembly and activation of NLRP3 inflammasomes, and promote the secretion of IL-1β, which ultimately induces granulopoiesis (22). A high number of neutrophils serves as a risk factor for adverse cardiovascular outcomes after myocardial infarction revascularization (23). In addition, neutrophils release neutrophil extracellular traps (NETs), which are complex network structures composed of DNA, histones, and granule proteins (24). It has been demonstrated that during MIRI, NETs in the myocardium exacerbate endothelial damage, activate the coagulation cascade reaction, trigger platelet binding and erythrocyte capture, and provide a scaffolding for thrombus formation, thereby mediating microcirculation obstruction and exacerbating ischemic injury (25, 26). A randomized, double-blind, placebo-controlled clinical trial included 363 AMI patients after PCI treatment to evaluate the relationship between neutrophil count and infarct size as well as ejection fraction. The results showed that a high neutrophil count was an independent predictor of short-term and long-term adverse clinical outcomes (27). The neutrophil to lymphocyte ratio (NLR) is a novel inflammatory biomarker, and MI patients with high NLR have a higher risk of developing angina, thrombosis, heart failure, major cardiovascular adverse events, and all-cause mortality. It has greater clinical predictive value than a single white blood cell count (28).

Neutrophils play a dual role in reperfusion-induced injury. In addition to pro-inflammatory responses and tissue damage, neutrophils are involved in regulating the regression of inflammation, wound healing, and cardiac remodeling (29). By constructing a model of chronic MI, a study found that neutrophil depletion induced deterioration of cardiac function, increased myocardial fibrosis, and elevated levels of biomarkers associated with heart failure (30). Neutrophils facilitate cardiac healing and remodeling by promoting macrophage polarization towards reparative M2 macrophages that mediate inflammatory abatement, thereby effectively removing dead cells and promoting angiogenesis, as well as the accumulation of myofibroblasts and collagen (30, 31). Similar to macrophages, neutrophils in the infarcted myocardial region have pro-inflammatory N1 and anti-inflammatory N2 phenotypes, and the proportion of the N2 phenotype increases over time, exerting anti-inflammatory and repair functions (32). A recent study applied single-cell RNA sequencing (scRNA-seq) to reveal the heterogeneity and diversity of neutrophils during MIRI pathology (6). Research has found that Ym-1^hi^Neu increases one day after reperfusion, and this tissue-specific subset exerts cardioprotective effects by promoting macrophage polarization to an anti-inflammatory phenotype, which may be a key subset mediating the repair of reperfusion injury and prognostic improvement (6, 33). Targeting neutrophil subpopulations is a novel approach for treating MIRI.

#### 3.1.2 Macrophages

**Figure 4:**
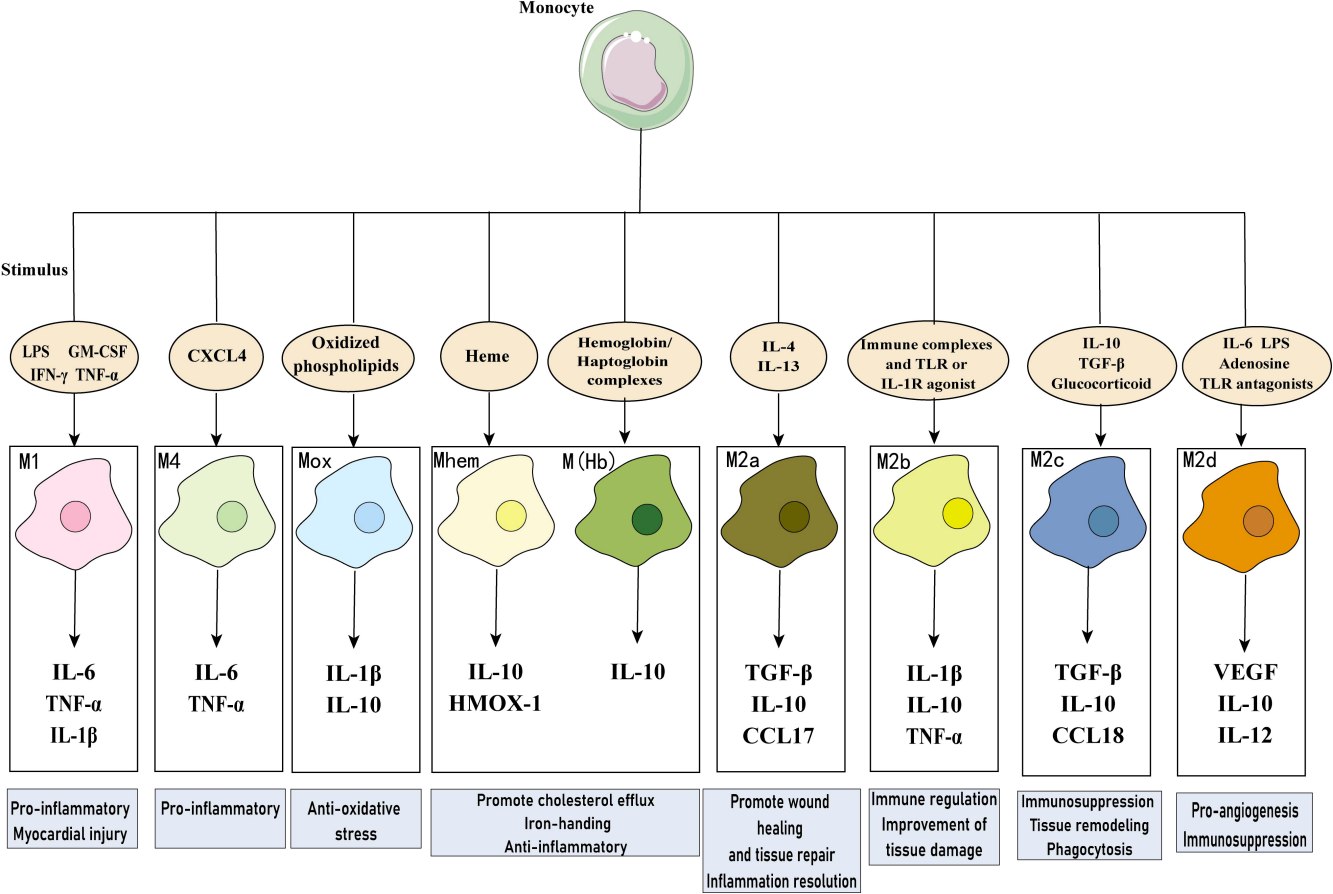
Macrophages in myocardial infarction. Diverse macrophage phenotypes exist in MI, which secrete multiple cytokines and perform different functions in response to different stimuli. LPS, lipopolysaccharide; GM-CSF, granulocyte-monocyte colony-stimulating factor; IFN, interferon; IL, Interleukin; COX-2, cyclooxygenase 2; TGF-β, transforming growth factor-β; VEGF, vascular endothelial growth factor; CCL17, chemokine (C-C motif) ligand 17; HMOX-1, heme oxygenase-1; CXCL4, C-X-C motif chemokine 4.

In the healthy heart, resident macrophages are the most abundant immune cell type, originated from yolk sac and fetal liver monocytes, which reside in the heart during embryonic development and have the capacity for self-renewal and repair (34). Resident macrophages play an important role in maintaining cardiac homeostasis, with functions such as anti-inflammation, removal of apoptotic cells, regulation of myocardial fibrosis, and promotion of tissue repair (35). After myocardial injury, recruited macrophages become dominant in both number and function. This type of macrophage is derived from circulating monocytes that produce inflammatory cytokines to amplify the inflammatory response and engulf necrotic tissue (36). Macrophages can be categorized simply based on function into pro-inflammatory M1 and anti-inflammatory M2 cells, with shifts in the dominant subpopulation occurring at different stages of repair process, but the transcriptional heterogeneity of macrophages in the infarcted area does not fully conform to this categorization (37, 38). M2 macrophages are divided into four subtypes based on stimulation: M2a, M2b, M2c, and M2d. In addition to M1 and M2 macrophages, there are special subgroups with different functions in MI, including M (Hb), Mhem, Mox, and M4 (39). These macrophage phenotypes have different cellular markers and functional characteristics, summarized in **Table 1**; **Figure 4**. In addition, researchers identified a new subtype of lipid-associated macrophages in MIRI, characterized by high expression of Spp1 and Trem2 genes, which are involved in regulating lipid metabolism and cardiac remodeling (40). Another study applied single-cell RNA sequencing technology to identify a unique S100a9hi macrophage population that participates in activating aseptic inflammation during the acute phase. However, when the tissue environment changes to reparative, this macrophage population can transform into a reparative phenotype, promoting fibroblast activation, repairing damaged tissue, and increasing angiogenesis (41). It can be seen that there are diverse populations of macrophages in the infarcted heart, and even the same phenotype may play different roles at different stages.

**Table 1:** Classification and function of macrophages.

Classification	Cell marker	Stimuli	Secretory products	Function	Refs
M1	CD80, CD86, iNOS, TLR2, TLR4, MCH-II	LPS, GM-CSF, IFN-c,IFN-γ, TNF-α,	IL-1α, IL-1β, IL-6, TNF-α, COX-2, iNOS	Pro-inflammatory; myocardial injury	(35, 42, 43)
M2a	CD206,Arg1, FIZZ1,Ym1/2	IL-4, IL-13	TGF-β, IL-10, CCL5, CCL17, CCL22, CCL24	Promote wound healing and tissue repair; inflammation resolution	(35, 42)
M2b	CD86, MHC-II	Immune complexes and toll-like receptors or IL-1 receptor agonist	IL-10, TNF-α, IL-1β, IL-6	Immune regulation; improvement of tissue damage	(44–46)
M2c	CD163, CD206, MerTK	IL-10, TGF-β, glucocorticoid	IL-10, TGF-β, CCL18	Immunosuppression; tissue remodeling; phagocytosis	(47, 48)
M2d	CD86, CD206	IL-6, LPS, adenosine A_2A_ receptor agonists, TLR antagonists	VEGF, IL-10, IL-12	Pro-angiogenesis; immunosuppression	(46, 49)
M(Hb)	CD163, mannose receptor	Hemoglobin,haptoglobin complexes	IL-10, ferroportin	Promote cholesterol efflux; reduce intracellular iron and ROS; anti-inflammatory	(50) (51)
Mhem	CD163, ATF1	Heme	IL-10, HMOX-1	Promote cholesterol efflux; iron-handing; anti-inflammatory	(51, 52)
Mox	Nrf2, HMOX-1, Srxn1, Txnrd1	Oxidized phospholipids	IL-1β, IL-10	Anti-oxidative stress	(53, 54)
M4	CD206, MMP7	CXCL4	IL-6, TNF-α	Pro-inflammatory	(52, 55)

LPS, lipopolysaccharide; GM-CSF, granulocyte-monocyte colony-stimulating factor; IFN, interferon; MHC-II, major histocompatibility complex-II; IL, Interleukin; COX-2, cyclooxygenase 2; iNOS, inducible nitric oxide synthase; Arg1, arginase1; FIZZ1, transcription factor found in inflammatory zone 1; TGF-β, transforming growth factor-β; VEGF, vascular endothelial growth factor; ATF1, activating transcription factor1; CCL17, chemokine (C-C motif) ligand 17; HMOX-1, heme oxygenase-1; Nrf2, nuclear factor E2-related factor 2; Srxn1,sulfiredoxin 1;Txnrd1, thioredoxin reductase 1; MMP7, matrix metalloproteinase 7; CXCL4, C-X-C motif chemokine 4.

In the infarcted heart, macrophages participate as core cells in all three stages of the repair process following reperfusion injury (56, 57). In the early stage of injury, monocyte-derived macrophages are recruited and activated, releasing large amounts of ROS and inflammatory mediators, which are involved in initiating the inflammatory response, engulfing dead cells, and degrading the extracellular matrix. Subsequently, macrophages clear apoptotic cells and trigger an anti-inflammatory response, which promotes the activation of myofibroblasts and angiogenesis to provide oxygen and nutrients to the deposited granulation tissue. In this way, the repair process moves from the inflammatory phase to the proliferative phase. The maturation phase occurs a few months after the proliferative phase, at this time there are fewer immune cells in the injured area, the extracellular matrix (ECM) is remodeled, the ventricle undergoes morphological and functional changes and a mature scar is formed (20, 56). The contribution of macrophages in the maturation phase needs to be further investigated. Cardiac remodeling after MI depends largely on the regulation of the ECM, which is composed mainly of structural and nonstructural matrix cellular proteins and proteases (58). During the inflammatory phase, macrophages secrete fibrous mediators to initiate fibrosis, releasing matrix metalloproteinases (MMP) to degrade collagen, fibronectin, and other ECM components. The synthesis of ECM components is significantly enhanced during the proliferative phase, in which the massive deposition of collagen forms a mature scar (59). One study reported the transcriptome changes of macrophages in the first week after infarction. It was found that macrophages exhibit pro-inflammatory features on day 1, engage in phagocytosis, proliferation, and metabolic reprogramming on day 3, and exhibit pro-repair features on day 7, reflecting the inflammatory, proliferative, and maturation phases of early injury repair (60). During MIRI, the metabolic processes and energy pathways of macrophages are altered, transitioning from anaerobic glycolysis to oxidative phosphorylation, which leads to a shift from pro-inflammatory to reparative macrophages. Early targeting of glycolysis facilitates the attenuation of cardiac injury and the improvement of cardiac function (61). Macrophages play an indispensable role in the MIRI process, promoting inflammatory resolution and damage repair through phenotype transformation, but excessive activation and infiltration exacerbate cardiac damage and remodeling.

#### 3.1.3 Dendritic cells

In addition to neutrophils and macrophages, dendritic cells (DCs) also play an important role in the pathological process of MIRI. DCs, as the main antigen-presenting cells, are the bridge between innate and adaptive immunity and contain two subtypes, cDCs and pDCs. The cDCs in the myocardium can be further categorized into cDC1s and cDC2s (21). Some investigators have quantified the extent of DCs infiltration in infarcted hearts, patients with ruptured hearts have less DCs, less reparative fibrosis, and more macrophage infiltration in myocardial tissues compared to patients with unruptured hearts (62). DC depletion exacerbates post-infarction cardiac dysfunction and remodeling and is associated with inducing degradation of the extracellular matrix, accelerating myocardial fibrosis, affecting angiogenesis, and enhancing inflammatory macrophage recruitment (63). It can be seen that DCs possess an immune protective effect after MI, which is beneficial for the repair and healing of the heart. Tolerogenic DCs have demonstrated potential for clinical translation as a new therapeutic strategy by inducing systemic activation of infarction-specific Tregs and promoting the conversion of macrophages from pro-inflammatory M1 to reparative M2 subpopulations, which leads to an improvement in cardiac remodeling and cardiac function (64). However, DAMPs released from ischemic cardiomyocytes during reperfusion stimulate pDCs to produce type I interferon, amplifying inflammatory response and exacerbating reperfusion injury (65). Exosomes are nanoscale extracellular vesicles released by cells, containing large amounts of cytoplasmic proteins, lipids, and nucleic acids that mediate intercellular signal transduction and communication (66). Several studies have demonstrated that DC-derived exosomes can promote angiogenesis, activate CD4+ T cells and Tregs, and regulate macrophage polarization, thereby improving cardiac function and exerting cardioprotective effects in infarcted hearts (67–70). There are fewer studies related to DC-derived exosomes intervening in the pathological process of MIRI, but exosomes have been recognized to attenuate reperfusion injury. The underlying mechanism might be related to the activation of the TLR4 downstream signaling pathway (71, 72). Its cardioprotective effect deserves in-depth study in the future.

#### 3.1.4 Innate lymphoid cells

Innate lymphoid cells (ILCs) are a small family of immune cells, a subpopulation of leukocytes with lymphoid properties that do not express antigen-specific receptors (73). ILCs are broadly classified into ILC1, ILC2 and ILC3 subtypes. After the injury, ILCs respond rapidly and participate in the immune response, producing effector cytokines such as IFN-γ, IL-5, and IL-13 in a manner similar to that of memory T cells, activating and modulating the immune response (74). In the healthy heart, ILCs are undifferentiated, do not express ILC2-specific markers, lack ILC1 and ILC3 markers, and these cells remain quiescent under physiological conditions (75). Deng et al. constructed a myocardial necroptosis mouse model using adriamycin and applied flow cytometry to analyze ILC subpopulations in the heart, and the results showed that ILC2 is the predominant cardiac resident ILC subpopulation, with type 1 ILCs present in a small quantity and ILC3 being nearly absent (7, 76). After myocardial injury, cardiac fibroblasts express increased IL-33, which induces rapid expansion and activation of ILC2 as well as the secretion of IL-4 to promote inflammation and tissue repair processes (75, 76). Yu et al. (77) reported that ILC2 residing in pericardial adipose tissue expands after MI. The deficiency of ILC2 increases the accumulation of inflammatory monocytes and macrophages and impedes cardiac remodeling and the recovery of cardiac function, activation of ILC2 with low-dose IL-2 can reverse these changes. It can be seen that amplification and activation of ILC2 in damaged myocardium are beneficial. In addition, natural killer (NK) cells, the only known cytotoxic member of the ILC family, are found in small numbers in the heart. These cells modulate the immune response after MI, preventing myocardial fibrosis and promoting vascular remodeling, which are beneficial to cardiac healing (78).

### 3.2 Adaptive immunity

#### 3.2.1 T cells

Abundant evidence suggests that adaptive immune responses regulate cardiac remodeling after MI and that T cells play an important role in cardiac injury and repair processes. In a mouse model of permanent coronary infarction, CD4^+^ T cells, CD8^+^ cells, and γδ T cells progressively infiltrate the heart, reaching a peak at day 7, whereas prompt reperfusion therapy advances the peak to day 3, promotes early inflammation abatement, avoids persistent inflammation, and limits infarct size (79). Myocardial damage activates four main subsets of CD4^+^ T cells, including helper T cells, i.e., Th1, Th2, and Th17 cells, and Tregs, with Th1 and Tregs being the major subsets (21). Th1 cells play a complex role in MIRI by inducing cell-mediated immune responses to combat infections and diseases, contributing to necrotic tissue clearance and damaged tissue recovery (80). But they are also associated with pathological events such as exacerbation of cardiomyocyte apoptosis, inhibition of myocardial fibrosis, enlargement of the damaged area and cardiac rupture (80). Th2 cells mainly regulate humoral immunity and secrete cytokines such as IL-4, IL-5, and IL-13, which play a crucial role in decreasing reperfusion injury, promoting myocardial fibrosis, inhibiting excessive inflammatory responses, and regulating immune homeostasis (80). Regulating the functional balance of Th1 and Th2 cells may be a potential therapeutic approach for MIRI. Th17 cells produce or induce the expression of pro-inflammatory cytokines such as IL-6, IL-1β, and IL-17, which exacerbate the inflammatory response and cause greater damage to cardiomyocytes, and also produce matrix metalloproteinases or proteoglycans to promote matrix remodeling after myocardial injury (80–82). In contrast to pro-inflammatory Th17 cells, Tregs are a specialized subpopulation of T cells that negatively regulate the immune response. Tregs mediate immunosuppression and immune tolerance, control the inflammatory response, maintain immune homeostasis, and help attenuate cardiac injury caused by reperfusion. Treatment with Tregs reduces cardiomyocyte death, promotes angiogenesis, and induces an anti-inflammatory environment, which in turn improves cardiac function and enhances the outcome of cardiac repair (83). In contrast, Tregs dysfunction exacerbates MIRI, enlarges infarct size, and deteriorates cardiac function, and pathological findings reveal more pro-inflammatory cells infiltration, increased cardiomyocyte apoptosis, and severe fibrosis (84). CD8^+^ T cells are also recruited and activated after myocardial ischemic injury, promoting cardiomyocyte death and leading to enhanced inflammation and decreased cardiac function, and their pathogenic role in the pathological process of MIRI has been demonstrated (85, 86). γδT cells are a special subset of T cells that perform innate immune functions, connecting innate immunity with adaptive immunity. These cells can rapidly produce large amounts of the pro-inflammatory cytokine IL-17A, which promotes cardiomyocyte apoptosis and neutrophil infiltration, ultimately exacerbating reperfusion injury (87, 88).

#### 3.2.2 B cells

B cells are ubiquitous in the naïve heart and represent a subpopulation of circulating B cells found in the cardiac microvascular system, mainly derived from bone marrow. The vast majority of B cells adhere closely to the vascular endothelium and only a few cross the endothelium into myocardial tissue. After injury occurs, mature B cells will be recruited to the damaged myocardial area, playing an important regulatory role in adverse cardiac remodeling (7, 89). After MI, the number of B cells infiltrating into the infarcted area reaches its peak on day 5, thereby mediating an immune response. Depletion of these cells significantly reduces the inflammatory response, decreases monocyte recruitment, limits the area of post-ischemic injury, prevents adverse ventricular remodeling, and improves cardiac function (89, 90). A study revealed a potential mechanism by which splenic marginal zone B cells regulate cardiac remodeling after MI, possibly through activation of the miR21/HIF-1α signaling pathway and upregulation of the expression of chemokine CCL7, which in turn promotes infiltration of inflammatory monocytes and adverse cardiac remodeling (91). If splenic B cells isolated from an infarcted mouse model are transferred to an atherosclerotic mouse model, they will increase IgG accumulation in plaques, expand the lesion area and accelerate the progression of fragile plaques (92). Targeting B-cell activation and antibody production constitutes a potential strategy for preventing recurrent cardiovascular events. Moreover, leukocyte infiltration and collagen deposition can be reduced by blocking the binding of lgM to ischemia-associated antigens in the heart, effectively alleviating MIRI (93). However, in addition to antibody production, some cytokines produced by B cells may allow them to play a beneficial role in myocardial infarction and reperfusion. For example, Wu et al. (94) found that pericardial adipose tissues are enriched with CD5^+^ B cells producing the anti-inflammatory cytokine IL-10. These cells expand and accumulate in ischemic hearts after MI, facilitating inflammatory regression and reducing myocardial injury. Thus, the roles of different B cell subpopulations, as well as the antibodies and cytokines they produce during myocardial injury are complex. The function of B cells in ischemic cardiovascular disease needs to be further investigated.

### 3.3 Crosstalk between different immune cells

After AMI, a large number of innate and adaptive immune cells are present in the injured area, which can interact with each other and jointly regulate the repair and remodeling of the infarcted heart. Macrophages, as one of the most active immune cells in the repair process of the damaged heart, can crosstalk with a variety of immune cells, such as neutrophils and T cells, which contribute to cardiac remodeling after AMI (36). Crosstalk between macrophages and neutrophils may be associated with IL-4, which down-regulates pro-inflammatory genes in neutrophils, up-regulates anti-inflammatory genes in macrophages, and enhances macrophages phagocytosis of neutrophils, inducing a more rapid reduction in inflammation (95). MMP-12 produced by Ly6C^low^ macrophages induces a more rapid decrease in inflammation by attenuating the chemokines CXCL1, CXCL2, and CXCL5 activity to inhibit neutrophil infiltration in the infarcted heart, thereby promoting wound healing (96). Depletion of resident macrophages in MIRI can alter macrophages crosstalk with other immune cells, inducing pro-inflammatory features of neutrophils, which are detrimental to cardiac remodeling (97). In addition, neutrophil apoptosis in the injured region can promote macrophage polarization towards a pro-repair phenotype, which in turn mediates the process of inflammatory abatement and cardiac repair (98). Neutrophils not only interact with macrophages but also exert a dual regulatory effect on adaptive immunity. On the one hand, neutrophils can activate and induce the proliferation of T cells and the secretion of cytokines, and regulate the activation of B cells, and on the other hand, neutrophils can inhibit adaptive immune responses to prevent excessive activation (31). Macrophages, as important antigen-presenting cells, phagocytose and process dead cells and cellular debris for presentation to T cells, initiating an adaptive immune response, which is associated with pathological remodeling of the infarcted heart. Cytokines secreted by T cells influence macrophage polarization. Th1 subpopulation of CD4^+^ T cells promotes macrophage polarization towards the M1 phenotype, while Th2 and Tregs promote macrophage polarization towards the M2 phenotype (99). Exosomes are key mediators of intercellular crosstalk that modulate the immune response after MI and maintain cardiac function through interactions between immune cells or between immune cells and cardiomyocytes (100). Exosomes may be associated with crosstalk between T cells and macrophages. DCs-derived exosomes have been found to activate Tregs, which in turn modulate macrophage polarization towards the M2 type to protect cardiac function in the infarcted heart (69). ILCs reflect the cytokine production and function of T cells, which can rapidly respond to injury and exert regulatory effects on T cells through MHC II-mediated antigen presentation or by regulating DCs (74). The crosstalk among ILCs, T cells, and DCs constitutes a complex network. Cardiac B cells can regulate different cell types, such as T cells, macrophages, and DCs, or interact with them to produce multiple cytokines (101).

Immune cells can interact with each other and crosstalk with cardiac parenchymal cells such as cardiomyocytes and fibroblasts. Large numbers of cardiomyocytes die in ischemic regions, and macrophages recognize and phagocytose dead cardiomyocytes via MerTk receptors, and increased CD47 expression in cardiomyocytes impairs the clearance of dead cells by macrophages (102). In addition to phagocytosis, macrophages also promote the proliferation and regeneration of cardiomyocytes (103). A recent study reported that SPP1hi macrophages, present in the early stages after MI, interact with reparative fibroblasts to promote collagen deposition and scar formation (104). Furthermore, exosomes derived from different immune cells can mediate fibroblast proliferation, migration, activation, and other processes that regulate myocardial fibrosis and remodeling after MI (105).

## 4 Cell death and immunity in MIRI

Various types of cell death occur during MI and reperfusion, such as apoptosis, necroptosis, ferroptosis, pyroptosis, and autophagy. There is an intricate relationship between cell death and the immune microenvironment. Apoptosis and necroptosis are the forms of cell death in the early stages of MIRI, while the predominant form of cardiomyocyte death in the later stages is ferroptosis, which is important for reperfusion-induced long-term cardiac injury (106). The Alox15-derived intermediate metabolite 15-HpETE has been reported to trigger cardiomyocyte ferroptosis by inducing mitochondrial dysfunction, leading to cardiomyocyte loss and exacerbating cardiac injury. The pharmacological inhibition of Alox15 reverses cardiomyocyte ferroptosis, thereby exerting cardioprotective effects (106). Zhang et al. (107) found that ferroptosis is involved in the pathogenesis of MIRI, and exosomes derived from bone marrow mesenchymal stem cells attenuate reperfusion-induced cardiac injury by inhibiting cardiomyocyte ferroptosis, and then improve cardiac function in mice. Ferroptosis is essential in the immune microenvironment and can interact with immune cells to promote the proliferation and activation of macrophages, T cells, and B cells, initiating an immune response by affecting the number and function of immune cells (12). The strong relationship between macrophages and ferroptosis has been demonstrated. On the one hand, macrophages can regulate iron metabolism and recognize and phagocytose iron-dead cells, on the other hand, ferroptosis induces macrophage polarization by affecting iron metabolism in macrophages and promotes macrophage recruitment to regulate immune responses (108–110). In addition, macrophages can undergo ferroptosis and participate in the pathological processes of several diseases, including atherosclerosis, tumors, and infectious diseases (108). It has been shown that extracellular vesicles released by macrophages remove excess transferrin-bound iron via surface transferrin receptors, attenuating iron overload and ferroptosis after MI, and thus exerting a protective effect against ischemia-induced cardiac injury (111). However, ferroptosis inhibition is not always beneficial to the heart. Research evidence suggests that iron-dead cardiomyocytes secrete IL-19 after MI, which promotes angiogenesis and modulates macrophage polarization towards the M2 type, favoring repair of damaged myocardium (112). In contrast, anti-iron death therapy inhibits angiogenesis and alters the immune response, further exacerbating cardiac injury (112).

For cell death after MI, it has been noted that the forms of cell death in ischemic myocardium include apoptosis, necroptosis, and pyroptosis, but do not include ferroptosis. Among them, pyroptosis is the most predominant form of programmed cell death, accelerating cardiac remodeling and dysfunction (113). During myocardial reperfusion, NLRP3 inflammasomes in macrophages activate and induce pyroptosis. Dying macrophages release more inflammatory cytokines, increase immune cell infiltration, promote damage to cardiac microvascular endothelial cells, and exacerbate microvascular dysfunction after revascularization (114). Stachyose has been reported to inhibit cardiomyocyte ferroptosis and macrophage pyroptosis, thereby significantly improving cardiac function and shrinking the infarcted area in MIRI mice (115). PANoptosis, a novel mode of programmed cell death, constitutes an integral part of the innate immune system and is driven by the innate immune sensors, pyrin, and ZBP1. This form of death exhibits key characteristics of apoptosis, necroptosis, and pyroptosis simultaneously, but cannot be explained by any one of these individual modes of death alone (116, 117). MIRI is an important inducer of PANoptosis, and combined inhibition of multiple forms of cell death exhibits a stronger cardioprotective effect than inhibition of one form of cell death alone, and targeting PANoptosis is a reliable way to ameliorate MIRI (117, 118). Neutrophils in infarcted hearts undergo a specific mode of cell death called NETosis and form NETs. Although it facilitates the clearance of pathogens, excessive NETosis amplifies the inflammatory response, exacerbates tissue damage and adverse remodeling, and may promote the onset and progression of heart failure (118). In addition to the above modes of cell death, autophagy can regulate the number and function of macrophages. Moderate macrophage autophagy can inhibit the inflammatory response, attenuate vascular injury, and promote regeneration of post-injury cardiomyocytes, which can help to ameliorate MIRI (119). Therefore, targeting different types of cell death in infarcted hearts may be a new therapeutic strategy for MIRI.

## 5 Targeting the immune system to intervene in MIRI

Immunotherapy is a promising treatment for ischemic heart disease. Immune cells, as the main executors of immune function, are used to readjust the immune microenvironment and thus regulate the body’s immune function to treat the disease. The CANTOS trial investigated the effects of a monoclonal antibody called Canakinumab in patients with previous MI and elevated high-sensitivity C-reactive protein. Canakinumab significantly reduced the recurrence of cardiovascular events by targeting the IL-1β innate immune pathway, demonstrating for the first time that immunotherapy can be beneficial for cardiovascular outcomes in patients (120, 121). This section will describe the progress of research related to immunotherapies that modulate the immune system to attenuate cardiac injury and promote tissue repair (**Table 2**).

**Table 2:** Targeted immune cell intervention in MIRI.

Immune cell	Therapeutic drugs or methods	Function	Potential mechanism	Refs
Neutrophil	Injection of MSC-Exo via tail vein.	Reduction of microvascular obstruction during reperfusion injury; improvement of cardiac function.	Inhibits neutrophil infiltration; reduces formation of NETs.	(122)
	Deliver VCAM-1 siRNA and dexamethasone using endothelial cell-targeting and ROS-ultrasensitive nanocomplexes.	Anti-inflammatory; alleviates MIRI; promotes cardiac recovery.	Restricts the recruitment of neutrophils.	(124)
	Delivery of adenosine generating enzymes CD39 and CD73 using hydrogels.	Long-term improvement of heart function.	Reduces neutrophil recruitment and activation.	(125)
	Injection of nanoparticles loaded with roscovitine.	Mediates inflammation resolution; protects heart function.	Induction of apoptosis in activated neutrophils; promotion of macrophage efferocytosis and M2-type polarization.	(98)
	Construction of engineered neutrophil apoptotic bodies.	Improves MI and promotes cardiac tissue regeneration.	Enhances macrophage phagocytosis and reprogramming.	(126)
Macrophage	Platelet membrane-modified extracellular vesicles.	Mediates cardiac repair by regulating the immune microenvironment.	Releases miRNAs; promotes the transformation of inflammatory M1 macrophages into reparative M2 macrophages.	(131)
	Lipid nanoparticle-delivered yREX3.	Reduces myocardial ischemic injury; limits the infarct area.	Silences Pick1 through DNA methylation; activates Smad3 to enhance phagocytosis.	(132)
	Engineered macrophage membrane-coated siRNA nanoparticles.	Reduces MIRI; improves cardiac function.	Improves the delivery efficiency of siRNA; reduces the level of S100A9.	(133)
	MiR-25-3p delivered by MSC-Exo.	Inhibits inflammatory response; improves MIRI.	Promotes the transformation of macrophage phenotype to M2 type.	(135)
	Natural melanin nanoparticles/alginate hydrogels.	Reduces inflammation; improves heart function.	Modulates macrophage polarization; scavenges ROS.	(136)
	Farrerol	Reduces inflammatory response and MIRI; inhibits apoptosis.	Inhibits the activation of NLRP3 inflammasomes in macrophages.	(137)
T cell	CAR-T cell therapy	Attenuates fibrosis; promotes functional recovery.	Targets fibroblast activation protein.	(139) (140)
	Systemic delivery of exogenous Tregs.	Reduces cardiomyocyte death; promotes cardiac repair.	Regulates the number and activity of specific macrophage subpopulations.	(83)
	Intrapericardial injection of MSC-Exo.	Mediates inflammatory resolution and cardiac repair.	Foxo3 activation; promotes the expression and secretion of Treg-inducing cytokines; activates Tregs.	(143)
	Injection of unrestricted somatic stem cells into infarcted myocardium.	Induces cardiomyocytes regeneration and left ventricular wall thickening; improves cardiac structure and function.	Regulates the migration and activation of T cells.	(144)
B cell	Empagliflozin	Improves cardiac function; prevents secondary myocardial injury.	Increases the number of bone marrow B cells.	(145)
	Rituximab	Limits the inflammatory response; improves heart function.	Depletes circulating mature B cells.	(148)

MSC-Exo, exosomes derived from mesenchymal stromal cells; NETs, neutrophil extracellular traps; ROS, reactive oxygen species; VCAM-1, vascular cell adhesion molecule-1; MI, myocardial infarction; CAR-T cell therapy, Chimeric antigen receptor-T cell therapy; Tregs, regulatory T cells.

### 5.1 Innate immunity

#### 5.1.1 Neutrophils

Neutrophils are the first type of leukocytes to infiltrate into the injured area after myocardial injury and play an important role in the immune system. Designing novel drugs and methods targeting neutrophils is crucial for limiting cardiac injury. Feng et al. (122) constructed a mouse model of MIRI and injected mesenchymal stem cell-derived exosomes (MSC-Exo) into the experimental group of mice via tail vein. MSC-Exo were found to significantly inhibit neutrophil infiltration and reduce the formation of NETs, attenuate the inflammatory response, and reduce microvascular obstruction during reperfusion injury, thereby improving cardiac function and exerting cardioprotective effects. The underlying mechanism may be that miR-199 packaged in MSC-Exo reduces the secretion of S100A8/A9 and inhibits the activation of NLRP3 inflammasomes, which in turn suppresses neutrophils. Clinical data have indicated that extensive microvascular obstruction is closely associated with higher neutrophil counts, and colchicine attenuates microvascular obstruction after MIRI through a similar mechanism, may be a potential agent to improve the prognosis of MI patients (123). A study developed endothelial cell-targeting and ROS-ultrasensitive nanocomplexes that synergistically limit neutrophil recruitment to the injured myocardium by co-delivering VCAM-1 siRNA and dexamethasone to exert an anti-inflammatory effect, thereby attenuating MIRI and promoting cardiac recovery (124). Studies have demonstrated that delivery of the adenosine-producing enzymes CD39 and CD73 using hydrogels reduces immune infiltration after MIRI, decreases neutrophil recruitment and activation, and facilitates long-term improvement in cardiac function (125). In addition, injection of nanoparticles loaded with roscovitine into a MI rat model to induce apoptosis of activated neutrophils can promote macrophage efferocytosis and M2 polarization, mediate inflammatory resolution, and protect cardiac function (98). Similarly, engineered neutrophil apoptotic bodies constructed by researchers can enhance macrophage phagocytosis and reprogramming, while initiating heme biosynthesis and production of anti-inflammatory bilirubin upon intracellular release, which in turn improve MI and promote cardiac tissue regeneration (126). Several studies have demonstrated that neutrophil membrane-encapsulated biomimetic nanoparticles can migrate more efficiently to the inflammation site, neutralize pro-inflammatory cytokines, modulate the immune microenvironment, and promote angiogenesis, thereby reducing cardiac damage and facilitating the repair of injured myocardium (127–129).

#### 5.1.2 Macrophages

As the most abundant immune cell population in the heart, the residence and polarization of macrophages are closely related to cardiac injury and repair. The development of drug delivery strategies that precisely target macrophages has potential therapeutic effect. Currently, studies have been conducted to improve macrophage targeting efficiency using tissue- or cell-specific promoters, surface-modified nanoparticles, and exosomes, but they are still in the early stages of development, with the ultimate goal of clinical translation (130). Li et al. (131) prepared platelet membrane-modified extracellular vesicles based on the membrane fusion method, which were delivered to the ischemic region using the ability of platelets to bind to monocytes. After escaping from the lysosomes of macrophages, they release miRNAs, promoting the transformation of inflammatory M1 macrophages into reparative M2 macrophages, and mediating cardiac repair by regulating the immune microenvironment (131). Researchers explored the potential mechanism by which lipid nanoparticle-delivered yREX3, a non-overlapping small Y RNA, exerts a cardioprotective effect, identifying macrophages as the target cells for its action. yREX3 silences Pick1 through DNA methylation, activates Smad3 to enhance phagocytosis, and then promotes cardiac tissue repair after MI (132). He et al. (133) prepared engineered macrophage membrane-coated siRNA nanoparticles. The surface-modified hemagglutinin ensured that the nanoparticles would not be damaged by lysosomal digestion, enabling them to effectively target the site of myocardial ischemia, improving the delivery efficiency of siRNA, reducing the level of S100A9, and improving the cardiac function of MIRI mice. Evidently, by constructing nanoplatforms coated with macrophage membranes on the surface, the precise targeting of ischemic areas and neutralization of pro-inflammatory cytokines can be achieved, thus improving drug delivery efficiency and therapeutic effects (134). Exosomes can protect the heart by regulating macrophage polarization. Studies have shown that miR-25-3p delivered by exosomes derived from bone marrow mesenchymal stem cells promotes a shift in macrophage phenotype to the anti-inflammatory M2 type, which in turn inhibits the inflammatory response and ameliorates MIRI (135). Some researchers have found that hydrogels prepared from natural biomaterials can regulate macrophage polarization and scavenge ROS through PI3k/Akt1/mTOR pathway to avoid cardiomyocyte apoptosis, which can be used for cardiac repair (136). Farrerol, a bioactive constituent of Rhododendron, can reduce the secretion of myocardial damage factors such as CK-MB, LDH, and NT-proBNP, and inhibit the release of IL-1β, IL-6, and TNF-α. It can also increase the level of antioxidant enzymes to alleviate oxidative stress, demonstrating powerful anti-inflammatory and antioxidant abilities. Farrerol has been reported to protect cardiomyocytes indirectly by inhibiting the activation of NLRP3 inflammasomes in macrophages and can be used as an immunomodulator for the treatment of reperfusion injury (137).

### 5.2 Adaptive immunity

#### 5.2.1 T cells

The modulation of immune responses mediated by different subpopulations of T cells can be used for cardiac injury treatment and repair. At present, the main T cell therapies for the treatment of MI mainly include Chimeric Antigen Receptor (CAR)-T cell therapy and the expansion and activation of Tregs. CAR-T therapy uses genetic engineering technology to modify T cells collected from a patient’s blood to express the CAR gene (138). These modified T cells are expanded *in vitro* and then infused back into the patient’s body, which is a precision-targeted therapeutic approach that has shown significant clinical efficacy in the treatment of cancer (138). Engineered T cells can also be applied in non-cancer therapies, and it has been found that adoptive transfer of specific CAR-T cells targeting fibroblast activation protein (FAP) attenuates fibrosis in the injured heart and promotes functional recovery (139). Rurik et al. (140) developed an *in vivo* method of generating CAR T cells, using CD5-targeted lipid nanoparticles to deliver therapeutic mRNAs encoding CAR receptors targeting FAP, in order to reprogram T cells *in vivo* to generate transient, potent CAR T cells. These cells eliminate pro-fibrotic cells in injured myocardium, thereby reducing myocardial fibrosis and improving cardiac function. Combining nanotechnology with CAR-T therapy is beneficial in enhancing efficacy and limiting toxicity, and may play an important role in the future development of CAR-T therapies (141). Tregs play a protective role in all phases of MI by suppressing inflammation and immune response. Tregs expansion may be a potential therapy for attenuating reperfusion injury and adverse remodeling and promoting cardiac healing (142). Researchers explored the effects and mechanisms of systemic delivery of exogenous Tregs on cardiac repair by constructing a mouse model of MI. They found that increasing the number of Tregs reduced cardiomyocyte death and promoted cardiac repair (83). Mechanistically, exogenous Tregs directly reduce pro-inflammatory Ly6C^Hi^CCR2^+^ monocytes/macrophages by expressing nidogen-1 and IL-10, and indirectly mediate the reduction of pro-inflammatory monocyte/macrophage subsets by regulating the number of CD8^+^T cells. Zhu et al. (143) found that intrapericardial injection of MSC-Exo could induce Foxo3 activation through PP2A/p-Akt/Foxo3 signaling pathway, which promotes the expression and secretion of Treg-inducing cytokines by antigen-presenting cells, and that activated Tregs mediate inflammatory resolution and cardiac repair. Intrapericardial injection of exosomes can be used as an immunomodulatory method for cardiac regeneration. In addition, injection of unrestricted somatic stem cells into the infarcted myocardium can regulate the migration and activation of T cells, form an inflammatory microenvironment dominated by T cells, induce cardiomyocyte regeneration and left ventricular wall thickening, and ultimately improve cardiac structure and function (144).

#### 5.2.2 B cells

B cells, as important immune cells, play a crucial role in cardiovascular disease by producing antibodies, secreting cytokines, and interacting with other cells. The cardioprotective effects mediated by empagliflozin may be achieved by induction of bone marrow-derived naïve B cells. It was found that empagliflozin treatment increased the number of bone marrow B cells, improved cardiac function, and prevented secondary myocardial injury in MIRI mice (145). These cardioprotective effects were further verified by tail vein infusion of bone marrow naïve B cells (145). Mechanistically, MI triggers the release of glucocorticoids, induces autophagy in NHE1-mediated bone marrow B cells, and inhibits the proliferation and differentiation of B cell progenitors. Tan et al. (146) demonstrated that murine neonatal cardiac B cells are essential for cardiomyocyte proliferation and cardiac regeneration, leading to improved cardiac function. However, in adult mice, cardiac B cells exacerbate inflammation and deteriorate cardiac function after myocardial injury. B-cell depletion emerges as a potential therapy to attenuate cardiac injury in adult mice. The potential reason may be that after cardiac injury, the proportion of protective B-cell populations with high expression of S100a6 and S100a4 is significantly reduced in cardiac tissues of adult mice compared with neonatal mice. Rituximab, a monoclonal antibody targeting B cells, selectively depletes B cells by binding specifically to CD20, a transmembrane protein on the surface of B cells (147). Intravenous infusion of different doses of rituximab in patients with acute ST-segment elevation MI within 48 hours after onset of the disease has been reported to be safe and feasible, effectively depleting circulating mature B cells and limiting the inflammatory response (148). Furthermore, targeting B-cell receptor signaling, B-cell survival, and B-cell and T-cell co-stimulation may be potential therapeutic strategies for cardiovascular disease, and further studies are needed for clinical translation (149). Additionally, vaccination to stimulate the production of protective antibodies by B cells offers a promising alternative approach that may be employed in the prevention and treatment of cardiovascular diseases in the future (149, 150).

## 6 Summary and discussion

MicroRNAs (miRNAs) are highly conserved single-stranded non-coding RNA molecules that play an integral role in the regulation of immune homeostasis as well as in the pathogenesis of MIRI. MiRNAs are involved in the regulation of cardiomyocyte apoptosis, inflammatory response, ventricular remodeling, and other pathological processes (151). MiR-146b-5p is up-regulated in the serum of infarcted mice, the application of antagomir to inhibit it significantly reduces cardiomyocyte apoptosis and cardiac fibrosis, promotes angiogenesis, and increases the number of reparative macrophages (152). Subsequently, this finding is further validated in a pig MI model, indicating that targeting immunomodulatory miRNAs may be a novel therapeutic strategy for ischemic heart disease (152). Exosomes, with their membrane structure, are natural carriers of miRNAs, protecting miRNAs from hydrolytic enzymes and ensuring their stable existence in the circulation. Exosomes of different cellular origins and the miRNAs they carry can mediate immune responses, inflammatory responses, cell migration, and intercellular communication, and participate in the repair process of ischemic injury by promoting angiogenesis, inhibiting cardiomyocyte apoptosis and myocardial fibrosis (153). Dai et al. (154) explored the mechanism of action of M2 macrophage-derived exosomes on MIRI. These exosomes carried miR-148a to the injury region after reperfusion and attenuated pyroptosis and myocardial injury by inhibiting the TLR4/NF-κB/NLRP3 inflammatory pathway. Researchers collected exosomes isolated from the serum of MI patients to determine their role in angiogenesis (155). They found that these exosomes may be released by cardiomyocytes and promote angiogenesis by down-regulating miRNA-143 to activate IGF-IR/NO signaling, thereby suggesting an anti-angiogenic effect of miRNA-143 (155). Gu et al. (156) devised engineered exosomes targeting cardiomyocytes and loaded miR302 into them, subsequently assessed their impact on MIRI *in vivo* and *in vitro*. The results demonstrated that these exosomes significantly attenuated cardiomyocyte apoptosis and inflammatory responses, leading to an improvement in cardiac function. It can be seen that miRNAs-related therapies have significant potential for the treatment of MIRI. Therefore, it is necessary to explore effective delivery systems that can prevent miRNAs from being degraded and accurately target and stably retain them at the infarction site, in order to facilitate miRNAs-mediated tissue repair.

Currently, various biomaterials such as nanoparticles, hydrogels, and bioengineered scaffolds are being applied alone or in combination with other bioactive molecules to modulate the immune microenvironment by acting on immune cells, cytokines, and chemokines, which in turn are used for tissue repair and regeneration (157). Nanosystems targeting multiple immune cells have created new opportunities for the treatment of cardiovascular diseases such as MI. The combination of nanotechnology with immunotherapy enables controlled and long-lasting immune modulation with the advantages of precise regulation and fewer side effects (158). A study evaluated the therapeutic effects of bilirubin nanoparticles on MIRI in mice and found that their cardioprotective effects were associated with attenuation of oxidative stress, apoptosis, and inflammation (159). Hao et al. (160) developed a novel injectable hydrogel with dual functions of ROS scavenging and NO release, which can regulate the ROS/NO imbalance after MIRI and attenuate cardiac injury and adverse remodeling, promote cardiac repair, and improve cardiac function. Some researchers have designed an injectable mitochondria-targeted nanodrug loaded-hydrogel (161). When injected into the MIRI myocardium, the hydrogel reduces the ROS level in the cardiac microenvironment, reduces cardiomyocyte apoptosis, and effectively recovers the mitochondrial and cardiac function, which can be used to attenuate reperfusion injury (161). Bioengineered scaffolds can be used for local cardiac biotherapeutics delivery. Decellularized scaffolds were applied to effectively deliver extracellular vesicles (EVs) derived from MSCs to the infarcted myocardium in a porcine model of MI. EVs were slowly released inside the scaffold, ensuring a locally effective dose, and exerting anti-inflammatory and immunomodulatory effects by promoting angiogenesis *in vivo*, reducing macrophage and T-cell infiltration, thereby promoting cardiac healing and improving cardiac function (162). Additionally, epicardial affixed devices, such as myocardial wraps and patches, mechanically stabilize the infarct site and can simultaneously provide a therapeutic effect or be applied in combination with bioactive materials for immunomodulation, providing a favorable environment for cardiac repair (163, 164).

Stem cell therapy has demonstrated great potential in treating MIRI by modulating the immune microenvironment of the infarcted heart through the paracrine secretome and cell membrane receptors, altering the recruitment and function of immune cells during cardiac repair (165). MSCs have emerged as an ideal stem cell type for the treatment of ischemic injury, and MSC-derived small EVs can modulate multiple pathological pathways in MIRI and show therapeutic advantages (166, 167). As immunosuppressants for myocardial repair, MSCs help reduce inflammation, oxidative stress, and myocardial fibrosis, but face the challenge of low cell survival rates for application (168). Systemic intravenous administration of human induced pluripotent stem cell-derived MSCs before transplantation has been reported to increase Tregs activation, reduce macrophage infiltration and apoptosis in infarcted areas, and improve the survival of transplanted cells in the myocardium after MI, promote neoangiogenesis and further improving left ventricular function (169). Recently, a functional biomaterial coating was used to encapsulate MSC spheroids (170). This coating protects MSCs from being cleared by the host immune system while providing ROS scavenging, stimulating pro-healing paracrine secretion of MSCs, and altering the pathological microenvironment through immune modulation, ultimately facilitating the repair of damaged myocardial tissue (170). Some researchers constructed a porcine MIRI model to investigate the effects of allograft MSCs delivered intravenously on reperfusion injury (171). The results demonstrated that intravenous injection of MSCs immediately after MIRI significantly attenuates microvascular obstruction and mitigates undesirable remodeling and inflammation by regulating immune cells (171). The safety and efficacy of MSC-based treatments for ischemic heart disease have been demonstrated in animal models, making them promising therapies for mediating cardiac repair and regeneration. However, there are discrepancies in the results of clinical trials, and the lack of standardized consensus and practical methods have limited their routine use in the clinic (172).

In summary, immune cells and various cytokines are involved in cardiac tissue repair and regeneration, which play pivotal roles in the pathological process of MIRI. Targeting the immune system can attenuate cardiac injury and promote damage repair. Currently, numerous animal studies have demonstrated the importance of immunotherapy in the treatment of ischemic heart disease by modulating the immune microenvironment. However, clinical trials have not achieved satisfactory results, and there are still challenges in translating basic experiments into clinical applications. Therefore, MIRI-related immunotherapies are still in an immature stage. In the future, preclinical experiments should be conducted using animal models with higher similarity to humans. Additionally, precise and effective drug-targeting delivery vectors need to be developed, and rigorously designed, high-quality clinical trials should be conducted to ultimately promote the clinical translation of immunotherapies.
